# Adaptive genetic mechanisms in mammalian Parp1 locus

**DOI:** 10.1093/g3journal/jkae165

**Published:** 2024-07-26

**Authors:** Yaroslava Karpova, Alexei V Tulin

**Affiliations:** Department of Biomedical Sciences, School of Medicine and Health Sciences, University of North Dakota, 501 North Columbia Road, Grand Forks, ND 58202, USA; Department of Biomedical Sciences, School of Medicine and Health Sciences, University of North Dakota, 501 North Columbia Road, Grand Forks, ND 58202, USA

**Keywords:** PARP1, poly(ADP-ribosyl)ation, PARylation, development, mouse knockout

## Abstract

Poly(ADP-ribose) polymerase 1 (PARP1) is a highly conserved nuclear protein in multicellular organisms that by modulating chromatin opening facilitates gene expression during development. All reported *Parp1* null knockout mouse strains are viable with no developmental anomalies. It was believed that functional redundancy with other PARP family members, mainly PARP2, explains such a controversy. However, while PARP2 has similar catalytic domain to PARP1, it lacks other domains, making the absence of developmental problems in *Parp1* mice knockouts unlikely. Contrary to prior assumptions, in our analysis of the best-investigated *Parp1* knockout mouse strain, we identified persistent mRNA expression, albeit at reduced levels. Transcript analysis revealed an alternatively spliced *Parp1* variant lacking exon 2. Subsequent protein analysis confirmed the existence of a truncated PARP1 protein in knockout mice. The decreased level of poly(ADP-ribose) (pADPr) was detected in *Parp1* knockout embryonic stem (ES) cells with western blotting analysis, but immunofluorescence staining did not detect any difference in distribution or level of pADPr in nuclei of knockout ES cells. pADPr level in double *Parp1 Parg* mutant ES cells greatly exceeded its amount in normal and even in hypomorph *Parg* mutant ES cells, suggesting the presence of functionally active PARP1. Therefore, our findings challenge the conventional understanding of PARP1 depletion effects.

## Introduction

Poly(ADP-ribose) polymerase 1 (PARP1), found abundantly within the nuclei of multicellular organisms, serves as a crucial chromatin remodeling factor and is highly conserved among species. Through the synthesis of poly(ADP-ribose) (pADPr) on other proteins or itself at gene promoters, PARP1 unwraps the chromatin and facilitates the gene expression across various organismal processes, especially in development (D’Amours et al. 1999; Amé et al. 2004; Ji and Tulin 2010; Boamah et al. 2012; Azarm and Smith 2020). Given its significant role in development, PARP1 or PARG deficiency causes developmental arrests in *Drosophila melanogaster* with 1 nuclear PARP protein fulfilling all poly(ADP-ribosyl)ating tasks (Tulin and Spradling 2003). The total depletion of PARP1 in embryos causes embryonic arrest, and totally knockout animal with maternally loaded PARP1 can develop until larval second stage. Hypomorph PARP1 mutants are arrested at late larval third stage and unable to complete the metamorphosis (Tulin et al. 2002; Tulin and Spradling 2003). The mutation in *Parg* gene that encodes the other key player of poly(ADP-ribosyl)ation pathway and the only enzyme that can degrades pADPr—similarly causes developmental arrest in *Drosophila* flies (Bordet et al. 2022).

**Fig. 1.**
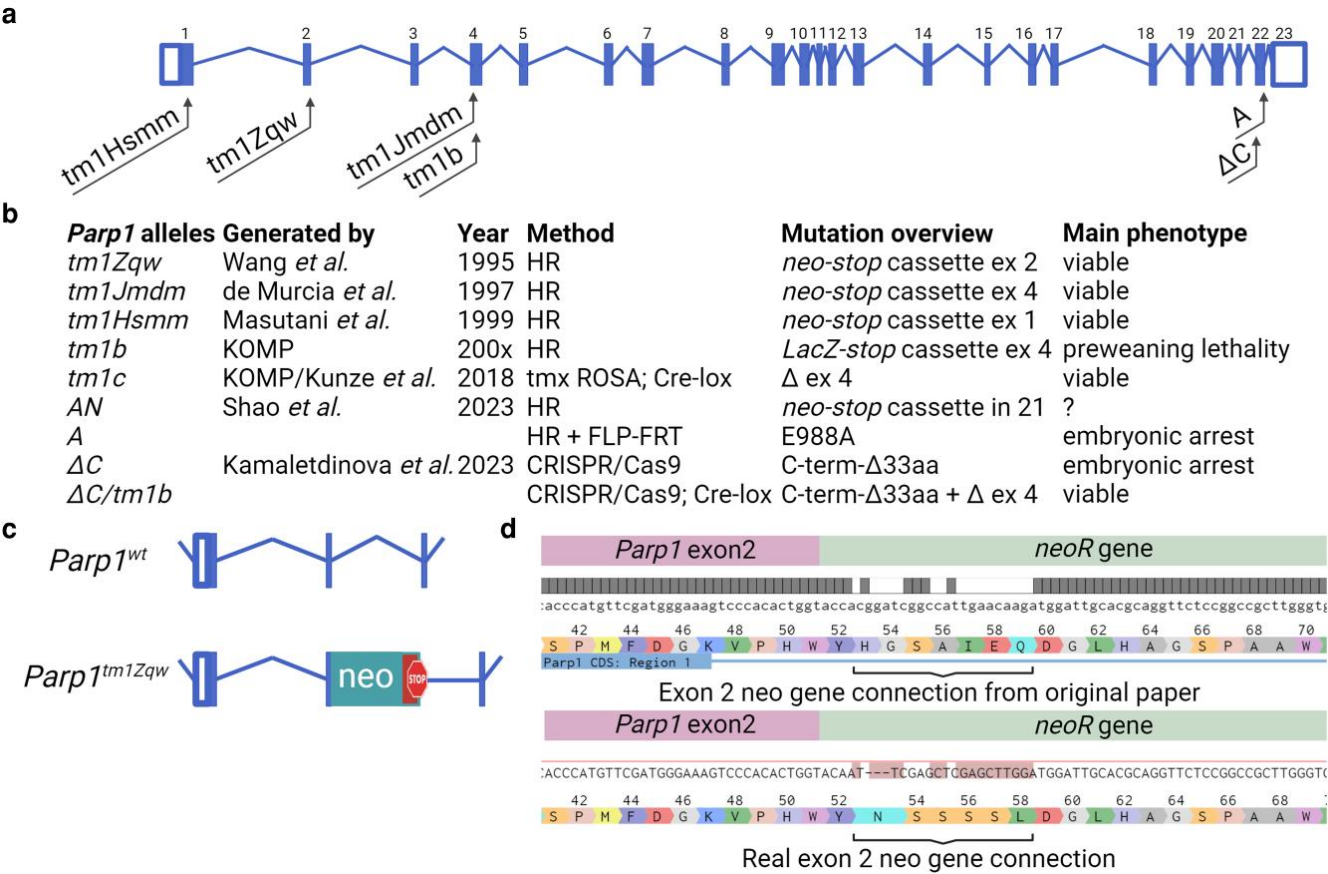
Targeting *Parp1* locus in mice. a) The model of *Parp1* gene. b) *Parp1* mutant mouse strains in whole animals. c) The knockout mice were generated by introducing neo cassette at the end of exon 2. d) The sequence of *Parp1* gene with introduced neomycin (*neo*) resistance cassette at exon 2. The sequence generated from description in the original paper and real sequence of *Parp1* exon 2 with neomycin resistance (*neoR*) gene connection generated from RNAseq experiment.

*Parp1* gene in mammals consists of 23 exons encoding a protein with 3 domains: DNA binding with zinc fingers (ZFs) I, II, and III, automodification (BRCT), and catalytic (WGR and PARP signature) domains (Fig. 1a–c) (Amé et al. 2004). PARP1 activity could be induced in 2 ways: by binding DNA through ZFs and by binding to histones H3 or H4 through C-terminal domains (Ikejima et al. 1990; Pinnola et al. 2007; Thomas et al. 2019). It was proposed that PARP1 activation through DNA is required for initiation of repair mechanisms when poly(ADP-ribosyl)ation serves as a histone remover and DNA repair enzyme attractor (Herceg and Wang 2001). On the contrary, gene activation is facilitated by interaction with H3 or H4 histones in nucleosomes with modified H2A histone variant that induce long-term PARP1 activation leading to chromatin remodeling and gene expression (Kotova et al. 2011; Thomas et al. 2019).

Three *Parp1* knockout mice lines were generated by different groups in 1995–1999 by *neo* cassette targeting exon 1 (Masutani et al. 1999), 2 (Wang et al. 1995), and 4 (Fig. 1) (de Murcia et al. 1997). Contradictory to studies in *Drosophila*, mice were viable with no developmental pathologies. However, residual poly(ADP-ribosyl)ation activity was still detected in these mice that was attributed to PARP2 activity (Shieh et al. 1998; Amé et al. 1999). However, direct evidence confirming that was not provided due to early embryonic lethality in double knockout mice (Ménissier de Murcia et al. 2003). Since 15 other PARP family members with lower expression were found in mammalian genomes, PARP2 is still the main candidate for functional redundancy with PARP1 (Amé et al. 2004). Nevertheless, the structural difference between PARP1 and PARP2, with PARP2 lacking all domains other than the catalytic one, make it unlikely that PARP2 can fully replace PARP1 functions.

Here, we focus on the most widely used *Parp1* knockout mice line generated by Wang et al. (1995) available through the Jackson Laboratory and demonstrated its hypomorph rather than total knockout nature, as PARP1 with an intact catalytic domain produces at RNA and protein levels.

## Material and methods

### Mice

129S-*Parp1^tm1Zqw/J^* mice (stock #002779) and C57Bl/J mice were obtained from Jackson Lab. 129S-*Parp^1tm1Zqw/J^* mice were transferred to C57Bl/J background by crossing for 7 generations. C57BL/6N-*A^tm1Brd^ Parg^tm2b(KOMP)Mbp^*/TcpMmucd mice were generated in the KOMP project and obtained from UC Davis (stock #048978-UCD) (Austin et al. 2004). All animal experiments were approved by the University of North Dakota Institutional Animal Care and Use Committee (IACUC). *Parg^tm2b(KOMP)Mbp^* mice were genotyped as described previously (Karpova and Tulin 2023). 129S-*Parp1^tm1Zqw/J^* mice genotyping was performed using regular PCR and the following primers:

oIMR6917 AGGTGAGATGACAGGAGATC

oIMR1473 CATGTTCGATGGGAAAGTCCC

oIMR1472 CCAGCGCAGCTCAGAGAAGCCA

### qRT-PCR

Total RNA was isolated from tissues with RNeasy kit according to the manufacturer’s protocol with genomic DNA elimination using g-column (Qiagen). Reverse transcription was performed with M-MLV reverse transcriptase (Invitrogen), and SYBR Green Master Mix (Applied Biosystems) was used for qPCR amplification. GAPDH level was used as a reference. The following primers were utilized:

mPARP1 F1 GGAGACCCGATTGGCTTAAT exon 20

mPARP1 R1 CCCTTGGGTAACTTGCTGATA exon 21

mPARP1 F2 CCTTGGTGGAGTACGAGATTG exon 14–15

mPARP1 R2 ACCTCGCTGAGGATAGAGTAG exon 15

mPARP1 F3 CTGCCTGGAGAAGATAGAGAAG exon 3

mPARP1 R3 GGTACCAGCGGTCAATCATA exon 4

mPARP1 F4 GGCCATCAAGAATGAAGGAAAG exon 4–5

mPARP1 R4 GCCTTCTCCAGCTTACTACTATC exon 5

mPARP1 F5 CAAGAAATGCAGCGAGAGTATT exon 1

mPARP1 R5 GGTACCAGTGTGGGACTTT exon 2

### Transcriptome analysis

RNA was isolated as for qRT-PCR. The quality of RNA was assayed with Bioanalyzer, and RNA samples ranged from 9.9 to 10 were considered for subsequent workflow. Library preparation and sequencing were performed at Novogen. RNA was enriched by poly(A) selection; library was prepared with Ultra II RNA library kit (New England Biolabs) and sequenced on NovaSeq 6000 (Illumina); 150 bp paired end sequencing reads were transferred to CLC Genomics Workbench v12.0, trimmed and aligned to GRCm38 mm10 mouse genome with standard parameters. Trimmed mean of M values normalization and gene expression analysis were performed. Aligned reads for *Parp1* gene genomic locus were selected for presentation. To retrieve sequences of expressed *Parp1* and *neo* cassette, their sequences were blast against all reads generated in RNAseq experiment and assembled.

### PCR analysis

cDNA synthesis was performed as for qRT-PCR and PCR performed using Hot Start Taq 2× Master Mix (New England Biolabs) and the following primers:

mPARP1 F8 GAGAGGCTTTATCGAGTGGAGT exon 1

mPARP1 R5 GGTACCAGTGTGGGACTTT exon 2

mPARP1 R3 GGTACCAGCGGTCAATCATA exon 4

The band corresponding exon 1–exon 4 amplicon was extracted with Gel extraction kit (Qiagen), and Eton Bioscience performed Sanger sequencing.

### Western blotting

Western blotting was performed as described previously (Johnson et al. 2022). Tissue and cell lysates were normalized on protein level and separated on 14% SDS-PAGE (Thermo Fisher) and transferred to nitrocellulose membrane (Thermo Fisher). The blots were blocked with 5% nonfat milk (Rockland) and stained overnight at 4°C with primary antibodies against pADPr (1:1000, sc-56198, Santa Cruz), β-actin (1:5000, A2228, Millipore Sigma), β-tubulin (1:20.000, B512, Sigma), PARP1 (1:1000, ab6079, Abcam), PARP1 (1:1000, 9542T, Cell Signaling), PARP1 (1:1000, ab32138, Abcam), and PARP1 (1:1000, MCA1522G, Serotec), or with special reagent against pADPr (1:2000, MABE1031, Millipore Sigma), and corresponding secondary antibodies anti-mouse HRP (1:3000, G21040, Invitrogen) and anti-rabbit HRP (1:1500, Perkin Elmer) for 1 h at room temperature.

### ES cell line generation and culturing

E3.5 blastocysts were obtained from timed pregnant females. To eliminate the zonae pellucidae, blastocysts were subjected to incubation in EmbryoMax Acidic Tyrode's Solution (Millipore Sigma). Zonae-free blastocysts were plated on gelatinized culture 96-well plates (Nunc) in ES culture media: KO DMEM (Gibco), 15% KO serum replacement (Gibco), 1× nonessential amino acids (Thermo Fisher), 1× Pen/Strep (Thermo Fisher), 1× Glutamax (Thermo Fisher), 0.1 mM 2-mercaptoethanol (Millipore Sigma), 5 µg/mL insulin, 1 µM PD0325901 MEK inhibitor (Stemcell Technologies), 3 µM CHIR99021 GSK3 inhibitor (Stemcell Technologies), and 200 U/mL LIF factor (Millipore Sigma). Upon formation of ES-like colonies, they were dissociated into individual cells using Accutase solution (Innovative Cell Technologies) and passaged as P0. ES cell sex was carried out using standard PCR with primers SX_F (GATGATTTGAGTGGAAATGTGAGGTA) and SX_R (CTTATGTTTATAGGCATGCACCATGTA) as previously described (McFarlane et al. 2013), and male ES cells were taken into analysis. *Parg^29b^* ES cells were generated by CRISPR/Cas9 (Karpova et al. 2023). *Parp1 Parg* double knockout ES cells were generated from intercrossed *Parp1^tm1Zqw/1tm1Zqw^ Parg^tm2b/wt^* mice.

### Immunofluorescence staining

ES cells were grown on gelatinized ibidi slides (Ibidi), fixed with 4% paraformaldehyde (Thermo Fisher) for 15 min at room temperature, permeabilized with 0.3% Triton X-100 (Sigma), and blocked with 5% normal goat serum (Abcam). Primary staining with antibodies against PARP1 (1:1000, 9542T, Cell Signaling) and pADPr (1:500, sc-56198, Santa Cruz) or against pADPr with special reagent (1:2000, MABE1031, Millipore Sigma) was performed for overnight at 4°C. Secondary antibodies anti-mouse Alexa 488 (1:1000, A28175, Invitrogen) and anti-rabbit Alexa 568 (1:1000, A11011, Invitrogen) were used for 45 min at RT. TOTO3 (Thermo Fisher) was used as a DNA/RNA marker. Confocal images were taken on Leica DMi8 microscope. For PARP inhibition experiment, ES cells were grown in culture media supplemented with 10 µM rucaparib (Selleck) or DMSO as a vehicle for 24 h before fixing (Karpova et al. 2023).

### Statistics

Statistical analyses were done using 2-tailed Student's t-test. A *P*-value of 0.05 or less was considered significant.

## Results

### PARP1 expression persists in *Parp1* knockout mice

**Fig. 2.**
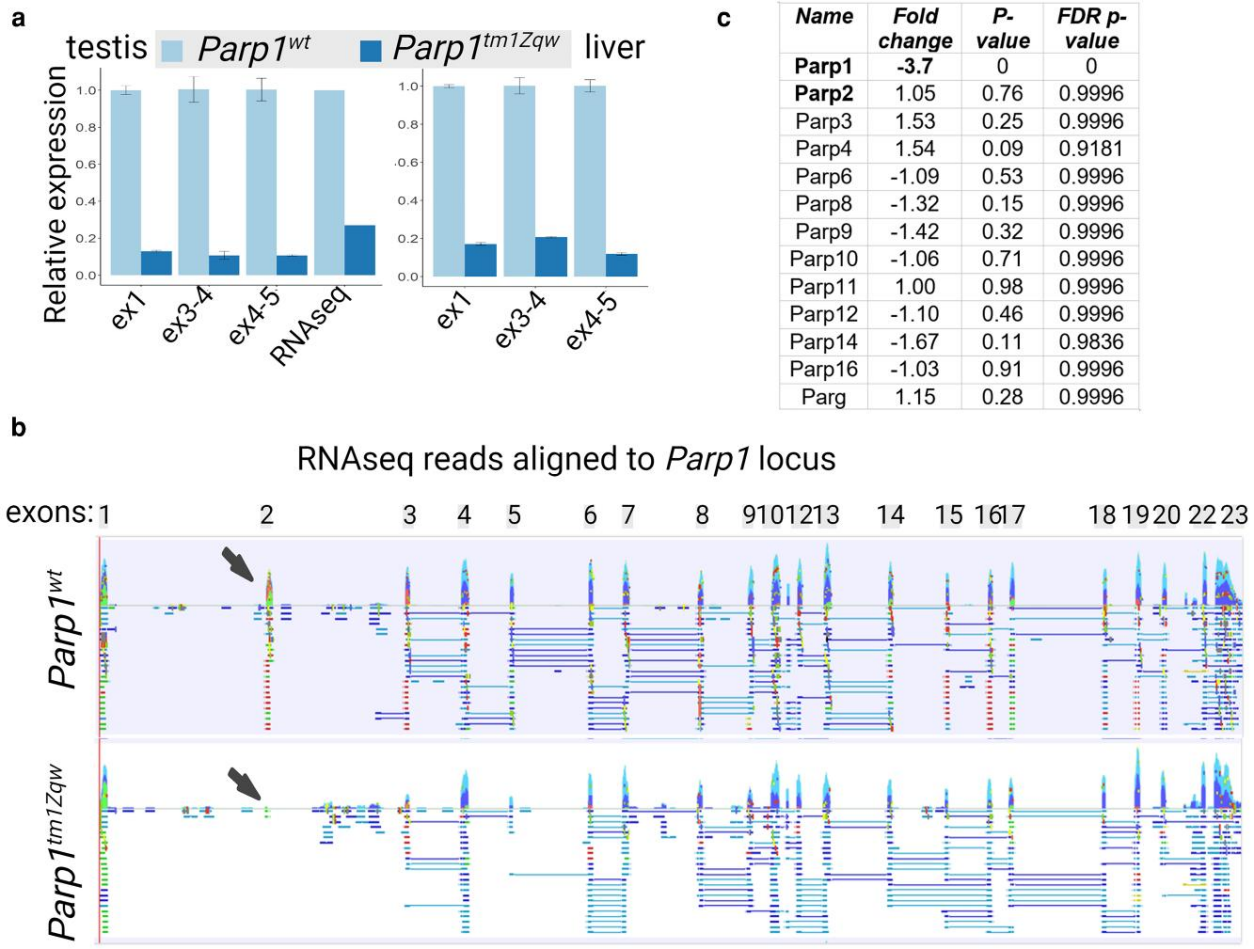
*Parp1* gene is expressed in *Parp1* knockout mice. a) qRT-PCR analysis with different sets of primers targeting first exon and exons after the neo cassette demonstrating the presence of *Parp1* mRNA in testis and liver of knockout mice. ex, exons of *Parp1* locus. b) *Parp1* gene complementary reads generated in RNAseq analysis in testis of control and *Parp1* knockout mice showing the presence of *Parp1* mRNA in both mouse strains. Exon 2 location is pointed by black arrow demonstrating the lack of exon 2–related reads in knockout mice. c) Differential expression analysis of *Parp* family members and *Parg* genes in testis of *Parp1* knockout mice.

Wang *et al*. created *Parp1* knockout mice by inserting a *neo-stop* cassette into exon 2 (Fig. 1b and c), resulting in the absence of detectable PARP1 protein products (Wang et al. 1995). The original article noted the presence of only 1 RNA transcript containing the first 2 exons of *Parp1* along with the *neo* cassette. To verify the insertions of *neo* cassette into exon 2 of *Parp1* gene, we performed RNAseq analysis and retrieved all *Parp1* and neomycin resistance gene corresponding reads. This analysis demonstrated the real sequence of connection between gene and inserted cassette that differs from published originally but confirmed the expression of neomycin resistance gene in frame with *Parp1* exons 1 and 2 (Fig. 1d). To confirm the lack of *Parp1* mRNA after the inserted cassette, we conducted qRT-PCR in selected tissues that expressed PARP1 at elevated (testis) and medium (liver) levels. Sets of primers after exon 3 detected 4–6-fold reduced but clearly detectable amount of *Parp1* mRNA in knockout mice (Fig. 2a). RNAseq of testis samples from control and knockout mice confirmed the presence of *Parp1* mRNA, with sequencing reads aligned to all *Parp1* exons except exon 2 (Fig. 2b). Differential expression analysis revealed a 3.7-fold decrease in *Parp1* expression in knockout testis, with no alterations for other PARP family members (Fig. 2c). These results indicate that despite the introduction of the *neo* cassette into exon 2 of *Parp1* gene, RNA transcription persists at an elevated level.

### Splicing of PARP1 in *Parp1* knockout mice

**Fig. 3.**
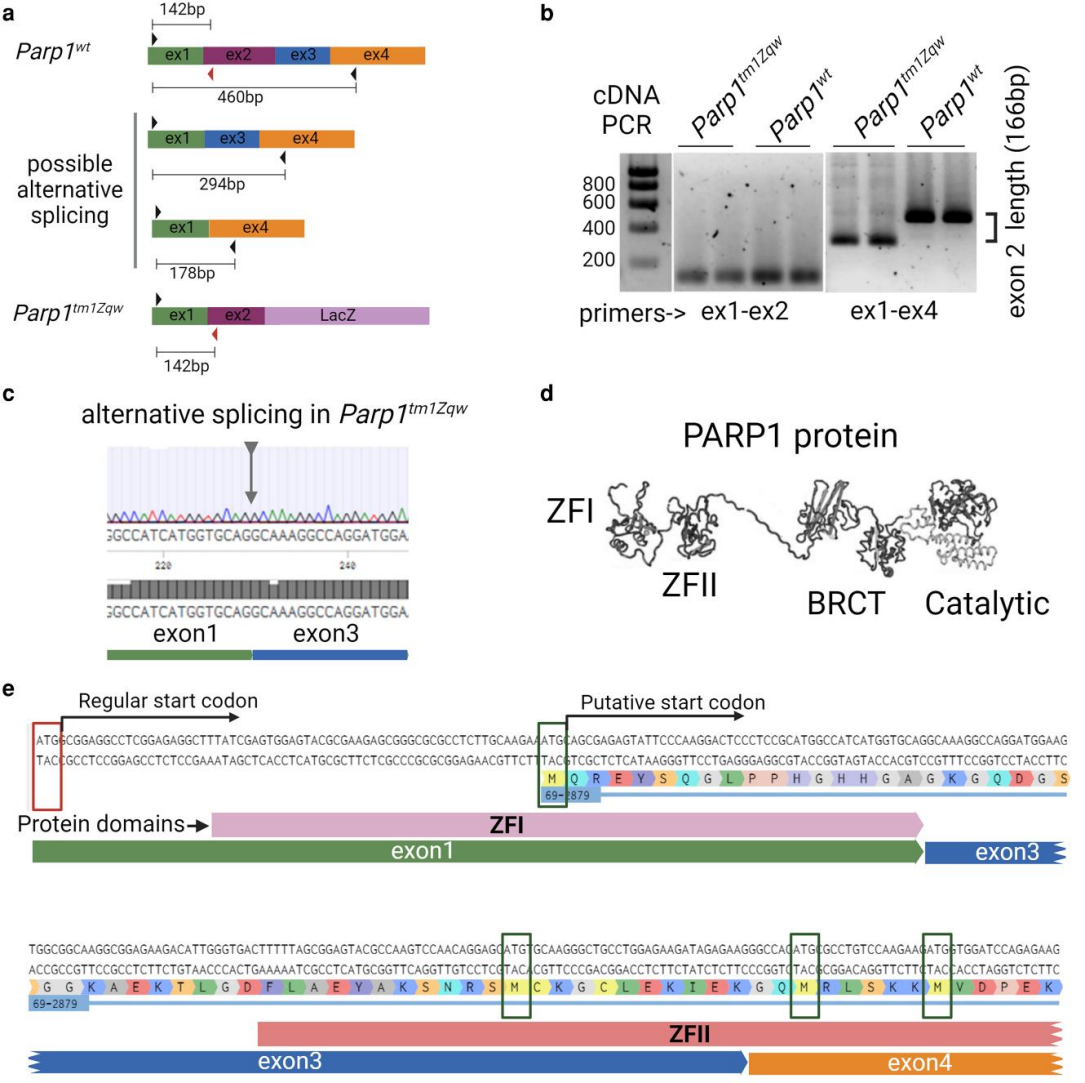
*Parp1* gene is alternatively spliced in *Parp1* knockout mice. a) Detection scheme of possible alternative splicing in Parp1 knockout mice. b) PCR amplification with testis cDNA from control and Parp1 knockout mice as a template and set of primers in exons 1 and 2 or exons 1 and 4. Agarose gel demonstrates the lower band amplified with primers in exons 1 and 4 from knockout mice that correspond the spliced out exon 2 in these mice. c) Sanger sequencing of PCR amplicon from a) demonstrating the splicing from exon 1 to exon 3 in knockout mice. d) The structure of PARP1 protein with ZFs, BRCT, WGR, and catalytic domains (Pinnola et al. 2007). e) The sequence of Parp1 cDNA with spliced exon 1 to exon 3 with classical (red, first codon) and putative (green, following codons) start codons, which could generate truncated version of PARP1 protein with disrupted ZFI or ZFI and II.

To test if the *neo* cassette is spliced out, we designed primers in different exons to assess the RNA length between them (Fig. 3a). Regular PCR on cDNA from tissue of control and knockout mice was performed using forward primer in exon 1 and reverse primers in exons 2 and 4. The analysis demonstrated the presence of a shorter PCR product for *Parp1* knockout mice, lacking exon 2 length, indicating splicing from exon 1 to exon 3 (Fig. 3b). Subsequent sequencing of the PCR product and RNAseq analysis of *Parp1* corresponding reads confirmed direct splicing from exon 1 to exon 3 of the *Parp1* gene (Fig. 3c).

This splicing event leads to a frameshift, rendering it incapable of generating the proper PARP1 protein. However, several downstream start codons in first and third exons of *Parp1* mRNA could potentially produce truncated versions of PARP1 protein without ZF I or ZFs I and II (Fig. 3d and e).

### *Parp1* knockout mice produce truncated PARP1 and retain highly efficient poly(ADP-ribosyl)ation activity

**Fig. 4.**
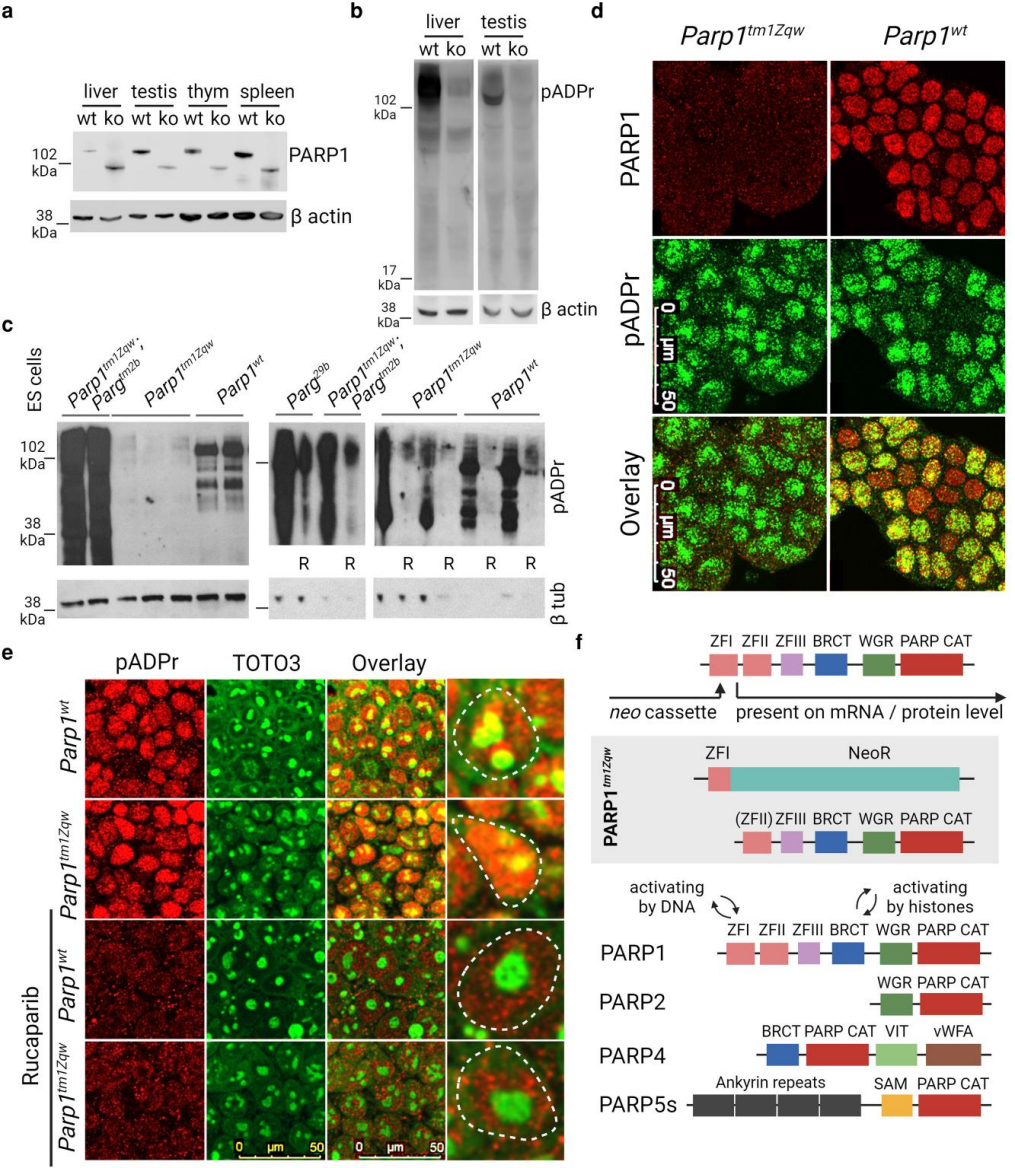
*Parp1* knockout mice express truncated version of PARP1 protein and produce elevated amount of pADPr. a) Western blotting analysis of different tissues of control and Parp1 knockout mice stained with antibodies toward whole PARP1 protein (Sero). The band with lower molecular weight is present in tissues of *Parp1* knockout mice. β-actin is shown as a loading control. b) Western blotting analysis of liver and testis from control and Parp1 knockout mice showing the presence but significantly lower amount of pADPr revealed by staining with antibody. β-actin is shown as a loading control. c) Western blotting of control, *Parp1* knockout, and *Parp1 Parg* double knockout ES cells stained with antibody against pADPr. The extreme accumulation of pADPr is noticeable in double *Parp1 Parg* knockout ES cells that can be reduced by rucaparib preincubation. β-tubulin is shown as a loading control. d) Immunofluorescence staining of ES cells established from control and *Parp1* knockout blastocysts with antibody against PARP1 (red, top panel) and pADPr (green, medium panel). No fluorescence signal is detected in *Parp1* knockout ES cells utilizing this antibody. However, the level of pADPr is the same in nuclei of control and knockout cells. e) Control and *Parp1* knockout ES cells were grown in the presence of 10 µM PARP inhibitor rucaparib for 24 h, and immunofluorescence staining was performed using reagent against pADPr (MABE1031) (red, left panel) and RNA marker TOTO3 (detects nucleoli in ES cell nuclei) (green, second from left panel). The reduced pADPr-related fluorescence intensity in rucaparib-treated cells demonstrate the successful PARP inhibition and specificity of pADPr staining. f) *Parp1* knockout and PARP protein family members with documented poly(ADP-ribosyl)ation activity and *Parp^1tm1Zqw^* locus.

The presence of *Parp1* mRNA in knockout mice prompted the investigation into PARP1 on a protein level in tissues high for PARP1 level (testis, spleen, and thymus) and medium (liver). From a tested panel of different anti-PARP1 antibodies in western blotting, one revealed a shorter 90 kDa protein version in all studied tissues of *Parp1* knockout mice (Fig. 4a). This indicates the translation of the mRNA into a truncated PARP1 protein in these mice.

As PARP1's primary activity is synthesizing pADPr, we examined pADPr level in knockout mice with western blotting analysis, confirming its presence at a lower level in *Parp1* knockout tissues (Fig. 4b) (Amé et al. 1999) and generated in the current study *Parp1* knockout ES (Fig. 4c). Surprisingly, immunofluorescence labeling did not demonstrate any difference between control and *Parp1* knockout ES cells (Fig. 4d and e). To verify the specificity of pADPr immunofluorescence and western blotting detection, we preincubated cells with PARP inhibitor rucaparib for 24 h. Decreased fluorescence and lower intensity band on western blotting were demonstrated in both control and knockout cells after rucaparib treatment confirming the correct pADPr detection (Fig. 4c and e).

Poly(ADP-ribosyl)ation is a very dynamic process, synthesized by PARP1. pADPr fast degrades by PARG protein, keeping PARP1 in unmodified and active state (Kotova et al. 2009). To estimate the intensity and efficiency of poly(ADP-ribosyl)ation in *Parp1* knockouts, we generated double *Parp1 Parg* knockout ES cells and performed the western blotting analysis. These ES cells demonstrated extreme accumulation of pADPr, which was even comparable to *Parg^29b^* hypomorph ES cells with catalytically inefficient PARG carrying 4 amino acid deletion in active center that could be successfully inhibited by rucaparib treatment (Fig. 4c) (Karpova et al. 2023). These results indicates that poly(ADP-ribosyl)ation remains highly efficient in *Parp1* knockouts.

## Discussion

We demonstrated that disrupted *Parp1* gene in the most utilized knockout mouse strain still expresses and produces truncated PARP1 possible via alternative splicing, which could explain the lack of developmental abnormalities in these mice.

In the original work on *Parp1* knockout mice, the presence of pADPr was demonstrated (Shieh et al. 1998). However, the absence of a truncated form of PARP1 protein in western blotting led researchers to search for other proteins with poly(ADP-ribosyl)ation activity, eventually discovering PARP2 protein and diverting attention away from investigating the potential leakage of *Parp1* expression (Amé et al. 1999). However, the difference in domain structure results in low probability of redundancy between PARP2 or other poly(ADP-ribosyl)ating protein and PARP1 (Fig. 4f).

Similarly, the other strain with disrupted exon 1 resulted to expression of PARP1 protein similar in size to the wild-type variant probably lacking ZF 1 (Masutani et al. 1999). These mice were claimed to be a total knockout, as it was believed that truncated PARP1 could not be activated (Ikejima et al. 1990). However, later research clearly demonstrated that PARP1 could be activated by histones in a ZF-independent manner (Fig. 4f) (Kotova et al. 2010; Thomas et al. 2019), and automodification with catalytic domains alone could successfully produce pADPr (Thomas et al. 2019). It was demonstrated both by successful activation of truncated PARP1 expressed from artificially created constructs and from PARP1 cleaved with caspase 3 in a cell-free system (Pinnola et al. 2007; Kotova et al. 2011; Thomas et al. 2014). The other *Parp1* knockout mouse strain with the disrupted exon 4 presents challenges for analysis due to difficulties in obtaining the strain (de Murcia et al. 1997). However, catalytic domain begins from exon 18, 18 kb away from exon 4, and the potential for truncated PARP1 protein expression remains highly plausible.

Interestingly, high preweaning lethality and incomplete penetrance were reported for *Parp1* knockout mice with disrupted exon 4 generated by KOMP project, suggesting the developmental abnormalities. The inconsistent results could be related to the remaining low expression of *Parp1* gene. Recently, this *Parp1* allele was recovered into conditional knockout mice, and Cre-lox-driven deletion in adult mice did not affect their viability (Kunze et al. 2019).

PARP1 is an abundant protein critical for development, and its diminished presence in *Parp1* mutant mice may still be sufficient for pADPr production and fulfilling some functions. Using double *Parp1 Parg* knockout ES cells, we demonstrated its remarkable ability to generate pADPr, suggesting remaining PARP1-related activity. However, the results of this work do not rule out the possibility that the presented PARP1 has altered or no activity and is functionally different from wild-type PARP1, with PARP2 or other pADPr-related enzymes responsible for the remaining activity. Studying truncated PARP1 for functionality and catalytic activity is complex, and it is challenging to obtain results that completely clarify the problem. We believe the most efficient method to address this is to create a new *Parp1* knockout mouse strain with complete deletion at all levels, which we are planning to do.

Recently, two more *Parp1* mutant strains were generated: with catalytically inactive PARP1 (Shao et al. 2023) and with catalytically inactive truncated PARP1 that is expressed at a very low level (Kamaletdinova et al. 2023). Both strains demonstrated early developmental arrest, similar to studies performed on *Drosophila* (Tulin and Spradling 2003). Moreover, introduction of the same catalytically inactive PARP1 in adult mice did not affect viability, highlighting the importance of PARP1 in the developmental process (Kunze et al. 2019). Notably, Shao *et al.* (2023) generated mice with disrupted intron 21 and mutated key catalytic amino acid, which was claimed as null allele. No evidence was demonstrated to prove it, and no phenotypes or viability information is available. Future publications could shed the light on these important questions.

Therefore, our study revealed the hypomorph nature of the commonly used *Parp1* knockout mouse strains. Our study strongly prompts a reassessment and potential reinterpretation of the effects attributed to PARP1 depletion in various cellular processes and pathological conditions. These unexpected findings advocate for a critical reexamination of the role and contributions of residual PARP1 activity in the physiological and pathological contexts previously ascribed solely to complete PARP1 loss.

## Data Availability

The data that support the findings of this study are openly available at the GEO database (accession no. GSE248429). Other data are available on request from the corresponding author.
